# Platelet-Derived Growth Factor Receptor and Ionizing Radiation in High Grade Glioma Cell Lines

**DOI:** 10.3390/ijms20194663

**Published:** 2019-09-20

**Authors:** Oana Alexandru, Ani-Simona Sevastre, Juan Castro, Stefan-Alexandru Artene, Daniela Elise Tache, Oana Stefana Purcaru, Veronica Sfredel, Ligia Gabriela Tataranu, Anica Dricu

**Affiliations:** 1 Department of Neurology, Faculty of Medicine, University of Medicine and Pharmacy of Craiova, Str. Petru Rares nr. 2-4, 710204 Craiova, Romania; 2 Department of Pharmacological Technology, Faculty of Pharmacy, University of Medicine and Pharmacy of Craiova, Str. Petru Rares nr. 2-4, 710204 Craiova, Romania; 3 Karolinska Institutet, Department of Oncology-Pathology, Cancer Center Karolinska, Karolinska University Hospital, Z1:00, 171 76 Stockholm, Sweden; 4 Department of Biochemistry, Faculty of Medicine, University of Medicine and Pharmacy of Craiova, Str. Petru Rares nr. 2-4, 710204 Craiova, Romania; 5 Department of Physiology, Faculty of Medicine, University of Medicine and Pharmacy of Craiova, Str. Petru Rares nr. 2-4, 710204 Craiova, Romania; 6 Department of Neurosurgery, “Bagdasar-Arseni” Emergency Hospital, Soseaua Berceni 12, 041915 Bucharest, Romania

**Keywords:** high grade glioma, radiotherapy, Platelet-derived growth factor receptor (PDGFR)

## Abstract

Treatment of high grade gliomas (HGGs) has remained elusive due to their high heterogeneity and aggressiveness. Surgery followed by radiotherapy represents the mainstay of treatment for HGG. However, the unfavorable location of the tumor that usually limits total resection and the resistance to radiation therapy are the major therapeutic problems. Chemotherapy with DNA alkylating agent temozolomide is also used to treat HGG, despite modest effects on survival. Disregulation of several growth factor receptors (GFRs) were detected in HGG and receptor amplification in glioblastoma has been suggested to be responsible for heterogeneity propagation through clonal evolution. Molecularly targeted agents inhibiting these membrane proteins have demonstrated significant cytotoxicity in several types of cancer cells when tested in preclinical models. Platelet-derived growth factor receptors (PDGFRs) and associated signaling were found to be implicated in gliomagenesis, moreover, HGG commonly display a Platelet-derived growth factor (PDGF) autocrine pathway that is not present in normal brain tissues. We have previously shown that both the susceptibility towards PDGFR and the impact of the PDGFR inactivation on the radiation response were different in different HGG cell lines. Therefore, we decided to extend our investigation, using two other HGG cell lines that express PDGFR at the cell surface. Here, we investigated the effect of PDGFR inhibition alone or in combination with gamma radiation in 11 and 15 HGG cell lines. Our results showed that while targeting the PDGFR represents a good means of treatment in HGG, the combination of receptor inhibition with gamma radiation did not result in any discernable difference compared to the single treatment. The PI3K/PTEN/Akt/mTOR and Ras/Raf/MEK/ERK pathways are the major signaling pathways emerging from the GFRs, including PDGFR. Decreased sensitivity to radiation-induced cell death are often associated with redundancy in these pro-survival signaling pathways. Here we found that Phosphoinositide 3-kinases (PI3K), Extracellular-signal-regulated kinase 1/2 (ERK1/2), or c-Jun N-terminal kinase 1/2 (JNK1/2) inactivation induced radiosensitivity in HGG cells.

## 1. Introduction

High grade gliomas (HGGs) are, according to the World Health Organization (WHO), a type of astrocytoma originating from the neuroglial cells of the central nervous system (CNS) [1].

In spite of general efforts made for many years to improve the therapies for HGG patients, their prognosis remains poor. However, in the last decades, as a consequence of the advances made in understanding the molecular basis of gliomas, some new agents have been tested. These new therapies include, among others, targeted therapies and immunotherapy [2]. In brain tumors, amplification of several tyrosine kinase growth factor receptors (GFRs) including Platelet-derived growth factor receptors (PDGFR), has been detected. Although the amplification of PDGF and PDGFR genes is not as common as EGFR, it still is encountered in about 16% of human gliomas [3].

Even since 1981, it has been established that PDGF and PDGFR are overexpressed in human glioma cell lines, but also in tumor surgical samples [4,5]. Actually, the high expression of this growth factor and of its receptors has been associated with a higher tumor grade [6]. The autophosphorylation of the receptor is followed by the activation of intracellular signaling pathways [7]. It was observed that the inhibition of PDGF and of PDGFRs is followed by a reduction of tumor proliferation, invasion, apoptosis, and angiogenesis. Nowadays, a number of PDGFR tyrosine kinase inhibitors like imatinib mesylate, or CP-673,451 have been tested but only with limited results in glioblastoma (GBM) therapy [8,9,10].

As hyperfractionated radiotherapy is a part of the standard of care for HGG patients, the improvement of this method continues to be very important. The adjustment of the doses plays an important role, but also other methods seem to be able to influence the radioresistance of malignant cells. Following these steps, it was observed that some anti PDGF/PDGFR molecules, like imatinib mesylate, have the ability to improve the sensitivity of tumor cells to radiotherapy [11]. The actual study is part of a series of investigations conducted in our group that were aimed to assess the effect of inhibition of GFRs on the response to gamma radiation treatment of solid tumors, including high-grade brain tumors in vitro. In this context, several small molecular inhibitors including AG336, SU1498, AG1024, LY294002, SB202190 etc., were used in our previously studies to analyze the involvement of GFRs and signal transduction in tumor cell radiosensitivity [12,13,14].

To date, the combination of radiation and PDGFR inhibitor AG1433 has not been evaluated in HGG enough. Here we investigate effects of AG1433, a PDGFR inhibitor, as a single treatment or combined with gamma radiation in HGG cells in vitro.

## 2. Results

### 2.1. The Effect of PDGFR Inactivation on HGG Cells

A usual irregularity in malignant brain tumors is the development of a putative PDGF-autocrine loop, generated by the co-expression of PDGFR and its ligand [15].

**Figure 1:**
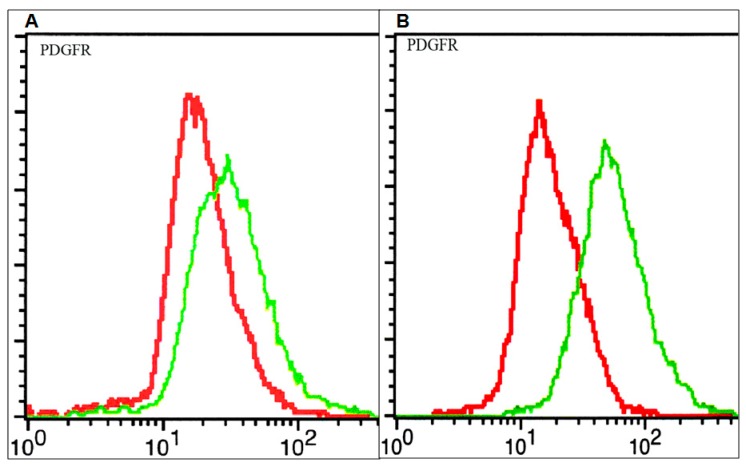
Membrane expression of Platelet-derived growth factor receptor (PDGFR) on glioblastoma cells: 11 high grade glioma (HGG) cells (**A**) and 15 HGG cells (**B**). Cells were stained with a plating efficiency (PE)-conjugated anti-PDGFR (green line) or a PE-labelled isotype control (red line) antibody and analyzed by flow cytometry as described in materials and methods.

The PDGF system was shown to be involved in different stages of brain tumor cell evolution (differentiation, proliferation, and apoptosis) [16]. We have previously shown that the HGG cell lines express different PDGFR amounts at the cell surface and also exhibit different susceptibility towards PDGFR inhibition [13]. In this study, in order to detect and quantify the PDGFR, flow cytometry was used. As can be seen in Figure 1, elevated amounts of PDGFR at the cell surface were observed in the 15 HGG cell line, while lower receptor levels were observed in the 11 HGG cell line.

In the last years, many active agents have been used to inhibit PDGFR [17]. We previously proved that AG1433 has a cytotoxic effect in several HGG cell lines, by using three different concentrations of active agent: 10, 20, and 30 µM [16]. For this current study, we used the same set of concentrations to evaluate the cytotoxic effect of the inhibitor in other two HGG cell lines, 11 and 15 HGG cells. The proliferation rates were evaluated at day 3 and at day 7.

**Figure 2:**
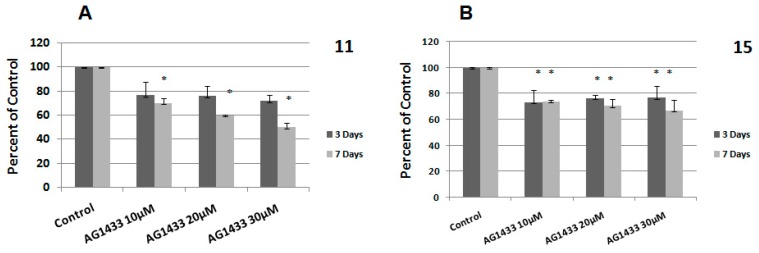
Effect of inhibition of PDGFR on glioblastoma (GBM) cells viability: 11 HGG cells (**A**) and 15 HGG cells (**B**) were seeded in 96-well culture plates (1–10 × 10^3^ cells/well) and treated with AG1433 for 3 days (grey bars) and for 7 days (black bars). Results are expressed as percentage of control and each experiment was repeated at least three times. Data are mean and standard error of three separate experiments. Data are reported as the mean ± SD (error bars). * *p* < 0.05 vs. untreated control cells.

We found that in the 11 HGG cell line, the treatment with 10 µM AG1433 induced 24% cytotoxicity after 3 days. After 7 days, the cytotoxic effect continued to increase up to 30% for the same concentration (Figure 2A). At 3 days after the treatment, a higher concentration of AG1433 (20 µM) maintained almost the same level of cytotoxicity in 11 HGG cells (25%). Prolonged treatment (7 days) was slightly more cytotoxic, but the result was not statistically significant (Figure 2A). As expected, greatest cytotoxicity was seen in 11 HGG cells treated with 30 µM AG1433, when the survival rate was reduced with approximately 29% after 3 days and with 50% after 7 days (Figure 2A).

In the 15 HGG cell line, the treatment with 10 µM AG1433 induced a slightly higher cytotoxicity after 3 days (26%) compared to that of the 11 HGG cell line (24%), using the same conditions. However, the cytotoxic effect decreased to approximately 23% and when concentrations of 20 and 30 µM were used (Figure 2B).

The cytotoxicity measured at 7 days in 15 HGG was lower than for 11 HGG, in the same experimental conditions. Thus, 7 days after the administration of 10 µM AG1433, induced 26% cytotoxicity, 20 µM AG1433 treatment induced approximately 30% cellular death, and the use of 30 µM AG1433 resulted in a 33% inhibition of cell viability (Figure 2B).

### 2.2. The Effect of Ionizing Radiation on HGG Cellular Viability

There are many studies, both in vivo and in vitro, which show that the majority of brain tumors are resistant to ionizing radiation treatment. Fractionation used in standard radiotherapy for glioma treatment comprises a total 45–60 Gy dose, administered in 1.8–2 Gy per fraction over a 5–6 weeks interval, while the most preclinical studies use a radiation dose that does not exceed 10 Gy [12,13,14,18,19,20].

**Figure 3:**
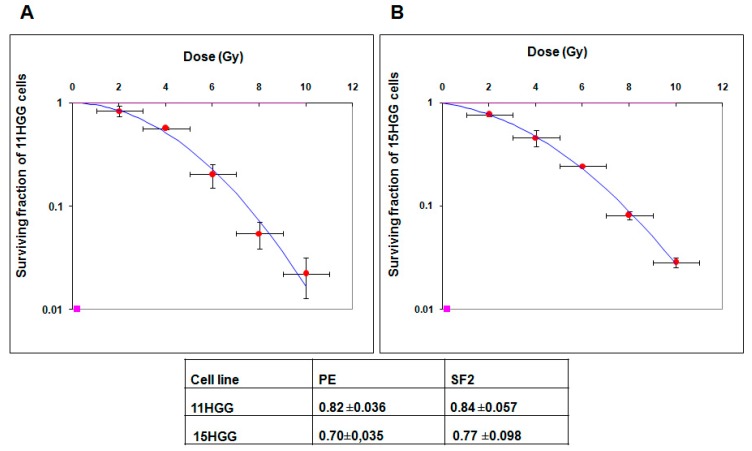
The effect of γ-radiation on colony-forming ability in 11 HGG (**A**) and 15 HGG (**B**) cells. Cells were plated (100–7500 cells/ 60-mm dish) and then were irradiated, using a ^137^Cs source. Twelve days later colonies were fixed and stained with crystal violet. Colonies that contained more than 50 cells were counted. Data are mean and standard error of two separate experiments.

In this study, we used doses ranging from 0 to 10 Gy to analyze the radiosensitivity of 11 and 15 HGG cell lines. By using MTT assay, that is based upon the cleavage of the yellow tetrazolium salt MTT to purple formazan crystals by metabolically active cells, we previously showed that 11 and 15 HGG cells are resistant to gamma radiation [21]. However, data from the literature showed that the most frequent method to analyze the response of cancer cells to radiation exposure is clonogenic assay, while MTT assay with a single-point detection of cell proliferation, is occasionally used. The clonogenic survival assay investigates cell capacity to proliferate endlessly, while maintaining its potential to give rise to a clone. In this case, the survival curve represents a relationship between the used radiation dose and the fraction of cells sparing their ability to form clones. For this reason, in this study we analyzed the radiosensitivity of 11 and 15 cell cultures by clonogenic assay. Survival curves depicted in Figure 3 were generated. In accordance with previous results, we found that both cell cultures were resistant to ionizing radiation. In our previous studies, by using MTT assay, we found that the 15 HGG line was a little more sensitive than 15 HGG exposed for 3 days to gamma-radiation at doses of 2, 4, 6, 8, and 10 Gy. However, unexpectedly, at longer exposure (10 days) at the same radiation doses (2, 4, 6, 8, and 10 Gy), the 11 HGG line turned out to be slightly more sensitive than the 15 HGG line [21]. It is noteworthy mentioning that clonogenic assay showed that 11 HGG was more radioresistant than 15 HGG, in terms of the survival fraction at 2 Gy (SF2). As shown in this figure, the average SF2 was 0.84 ± 0.057 for the 11 cell line and 0.77 ± 0.098 for the 15 cell culture.

### 2.3. The Effect of Combined Treatment on HGG Cell Viability

The effect of radiotherapy remains problematic, because the cell response to radiation treatment is influenced by many factors. The combination of radiotherapy with tumor specific agents was suggested to induce change or alteration in the tumor cells’ responses to the radiation [22]. Several studies indicated that radiation induces membrane growth factor receptors phosphorylation, leading to an increase in cell proliferation [23,24]. Therefore, we considered that radiation treatment should be done concurrently with inhibition of the receptor inactivation.

Here, we evaluated the effect of the PDGFR inhibition on sensitivity to radiation in 11 and 15 HGG cell lines. For this purpose, the 11 and 15 HGG cells were treated with 10, 20, or 30 µM AG1433 and irradiated with single doses of 2, 4, 6, 8, or 10 Gy ionizing gamma radiation.

**Figure 4:**
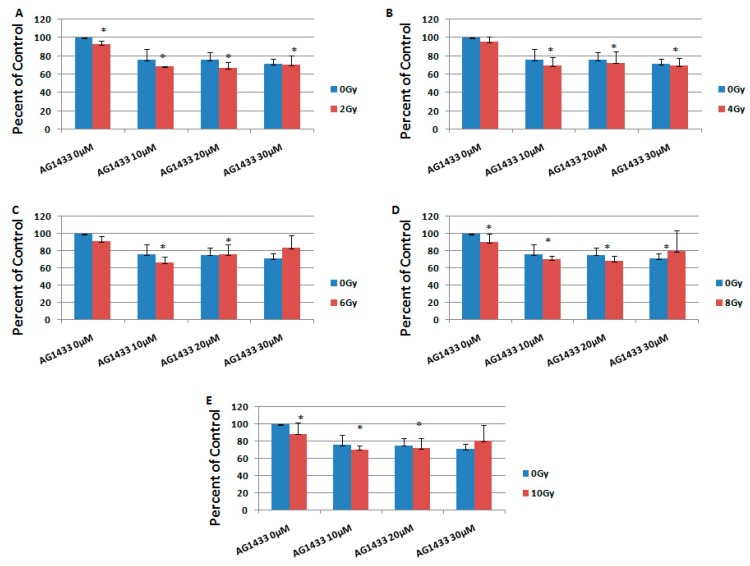
The effect of PDGFR inhibition on ionizing radiation response in 11 HGG cells, 3 days after the treatment. The cells were seeded in 96-well culture plates (5000 cells/well) then irradiated with 2 Gy (**A**); 4 Gy (**B**); 6 Gy (**C**); 8 Gy (**D**); 10 Gy (**E**) and treated with 10, 20, or 30 μM AG556. The cells were incubated for 3 days and cell viability was determined by MTT assay that is based upon the cleavage of the yellow tetrazolium salt MTT to purple formazan crystals by metabolically active cells. All results show the mean of three independent experiments ± SD (error bars). * *p* < 0.05 vs. irradiated cells.

**Figure 5:**
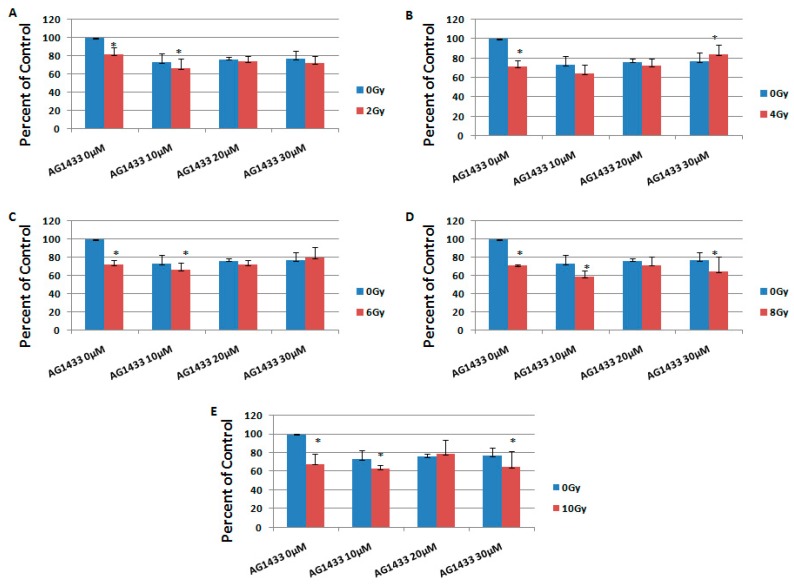
The effect of PDGFR inhibition on ionizing radiation response in 15 HGG cells, 3 days after the treatment. The cells were seeded in 96-well culture plates (5000 cells/well) then irradiated with 2 Gy (**A)**; 4 Gy (**B**); 6 Gy (**C**); 8 Gy (**D**); 10 Gy (**E**) and treated with 10, 20, or 30 μM AG556. The cells were incubated for 3 days and cell viability was determined by MTT assay. All results show the mean of three independent experiments ± SD (error bars), * *p* < 0.05 vs. irradiated cells.

It was revealed that the combination of AG1433 and radiation reduced the HGG cell viability, in a concentration-dependent manner (Figure 4 and Figure 5).

At three days after the treatment, in the 11HGG cell line exposed to 2 Gy radiation, the treatment with 10 µM AG1433 led to the highest cytotoxicity for the 20 µM AG1433 treatment (Figure 4A), while the highest cytotoxic effect of 4 Gy gamma radiation was after 30 µM AG1433 treatment (Figure 4B). The cytotoxic effect of 8 Gy radiation was 31.4% after 20 µM AG1433 treatment (Figure 4D), while for the same concentration of AG1433 combined with 10 Gy ionizing radiation, the cytotoxic effect reached only 27.8% (Figure 4E).

Also after three days, the treatment with 10 µM AG1433 induced a cytotoxic effect of 33.4% in 15 HGG cells irradiated with 2 Gy (Figure 5A), while combined with 4 Gy gamma radiation, it resulted in 35.3% cellular death (Figure 5B). The cytotoxic effect of 6 Gy radiation was of 33.3% when adding 10 µM AG1433 treatment, and maintained almost the same (27.6%) after treatment with 20 µM AG1433 (Figure 5C). The cytotoxic effect of 8 Gy radiation was 40.8% when combined with 10 µM AG1433 (Figure 5D), while using 10 Gy ionizing radiation combined with the same concentration of active ingredient led to 36.8% cytotoxicity three days after the treatment (Figure 5E).

In particular, the minimum reduction in cellular viability for the combined treatment was observed at 3 days post-treatment in the 11 HGG cell line (Figure 4C) for the combination of 30 µM AG1433 and 6 Gy irradiation (16.60%), whereas for the 15 HGG cell line, the cytotoxic effect was 20.03% for the same experimental conditions (Figure 5C). The minimum cytotoxic effect in the 15 HGG was 15.65% for the combined treatment of 30 µM AG1433 and 4 Gy irradiation 3 days after the treatment (Figure 5B).

At 3 days post-treatment, the maximum cytotoxic effect in 11 HGG cell lines was 33.15% for 10 μM AG1433 combined with 6 Gy irradiation (Figure 4C), whereas for the 15 HGG cell line, the highest cytotoxic effect was 40.84% for the same concentration of active principle, but combined with 8 Gy irradiation (Figure 5D).

**Figure 6:**
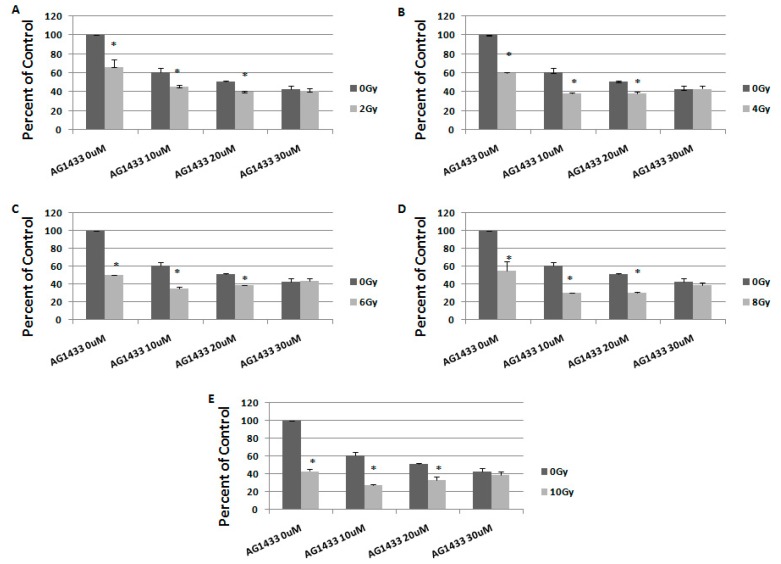
The effect of PDGFR inhibition on ionizing radiation response in 11 HGG cells, 7 days after treatment. The cells were seeded in 96-well culture plates (3000 cells/well) then irradiated with 2 Gy (**A**); 4 Gy (**B**); 6 Gy (**C**); 8 Gy (**D**); 10 Gy (**E**) and treated with 10, 20, or 30 μM AG556. The cells were incubated for 3 days and cell viability was determined by MTT assay. All results show the mean of three independent experiments ± SD, * *p* < 0.05 vs. irradiated cells.

**Figure 7:**
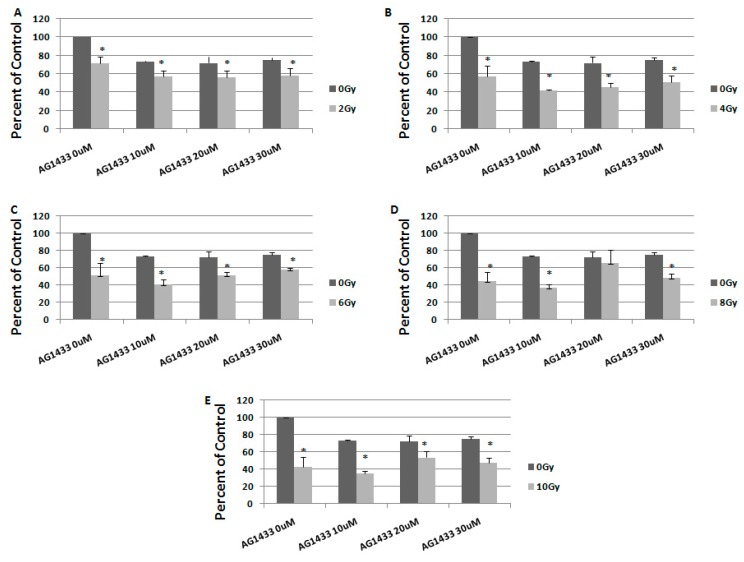
The effect of PDGFR inhibition on ionizing radiation response in 15 HGG cells, 7 days after treatment. The cells were seeded in 96-well culture plates (3000 cells/well) then irradiated with 2 Gy (**A**); 4 Gy (**B**); 6 Gy (**C**); 8 Gy (**D**); 10 Gy (**E**) and treated with 10, 20, or 30 μM AG556. The cells were incubated for 3 days and cell viability was determined by MTT assay. All results show the mean of three independent experiments ± SD, * *p* < 0.05 vs. irradiated cells.

To study the long-term effect of the treatment, we evaluated the response to combination therapy, 7 days after treatment (Figure 6 and Figure 7).

One week after the treatment, when 2 Gy irradiation was used, the maximum cellular death (59.9%) was observed for the combination with 20 µM AG1433, for the 11 HGG cell line (Figure 6A). When 11HGG cells were irradiated with 4 Gy, the treatment with 20 µM AG1433 led to to cellular death of 61.7% (Figure 6B), while the cytotoxic effect of 6 Gy gamma radiation was 61.2% when combined with 20 µM AG1433 (Figure 6C). The cytotoxic effect of 8 Gy radiation was 69.7% after treatment with 10 μM AG1433, one week after the treatment (Figure 6D) and the maximum citotoxic effect was observed for the combination 10 Gy ionizing radiation and 10 μM AG1433 (Figure 6E).

In 15 cells irradiated with 2 Gy, the treatment with 10 μM AG1433 led to a cellular cytotoxic effect of 43%, one week after the treatment (Figure 7A). By increasing the radiation to 4 Gy, for the same concentration of active ingredient, the cellular death reached 58% (Figure 7B), 59% when 6 Gy was used (Figure 7C), 63.6% when 8 Gy was used (Figure 7D) and 64.8% for the combination with 10 Gy irradiation (Figure 7E).

A maximum reduction of cellular viability in both 11 HGG (72.17%) and 15 HGG (64.78%) cell lines was observed at 7 days post-treatment for a concentration of 10 μM AG1433 combined with 10 Gy irradiation (Figure 6E and Figure 7E, respectively).

### 2.4. The Interaction between Combined Treatment with AG1433 and Ionizing Radiation in HGG

The interaction between two or more concomitant therapies can lead to sub-additive, additive, or synergistic effects. Therapies which when used together lead to the additive or synergistic effect are preferred.

As it can be seen in Table 1, for the 11 HGG cell line, of the 30 combinations, only 15 had an additive effect (50%) and a synergistic effect was not achieved in any of the attempted combinations.

When the 15 HGG cell line was used, 93% of the combinations (28 out of 30) had a sub-additive effect and only 7% (2 out of 30) had an additive effect (Table 2). We did not obtain a synergistic effect in any of the attempted combinations.

### 2.5. The Effect of Signal Transduction Inhibition on Radiation Response in HGG Cells

Growth factor receptors, including PDGFR, are auto-phosphorylated upon ligand binding, which results in the activation of two major intracellular pathways: PI3K/AKT and MAPK pathway. Radiation induced intracellular signaling has been found in many cancers and targeting these pathways can result in radiosensitivity [25]. To analyze the response of HGG cells to ionizing radiation after PDGFR downstream signaling inactivation, in we used LY294002 (a phosphatidylinositol 3-kinase (PI3K) inhibitor), PD 98059 (a selective, Erk1/2 inhibitor), and SP600125 (a selective inhibitor JNK1/2).

**Figure 8:**
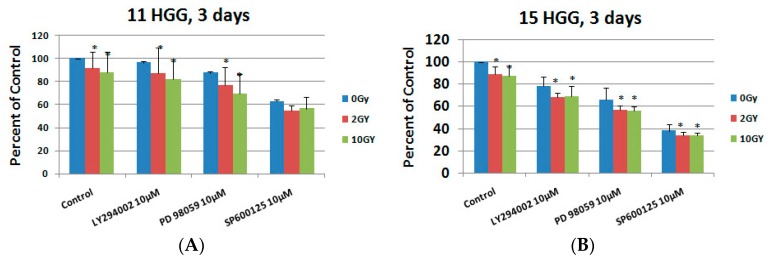
The effect of signal transduction inhibition on ionizing radiation response in 11 (**A**) and 15 HGG (**B**) cells. The cells were seeded in 96-well culture plates (5000 cells/well) then irradiated with 2 (red bar) and 10 Gy (green bar) and treated with 10 μM LY294002, PD 98059, and SP600125 and then cell growth was measured using MTT assays after 3 days. All results show the mean of three independent experiments ± SD, * *p* < 0.05 vs. irradiated cells.

At 3 days after the treatment, the cell survival rate decreased in the following order LY294002 > PD 98059 > SP600125, for all combinations, as can be observed in Figure 8A,B.

For the 11HGG cell line, the maximum cytotoxic effect (45.16%) was for the combination SP600125 10 µM and 2 Gy (Figure 8A), whereas for 15 HGG the maximum decrease in cellular viability rate (66.36%) was for the combination SP600125 10 µM and 10 Gy (Figure 8B).

For the LY294002 and PD 98059 used in combination with irradiation treatment, changes in the cell survival rate were observed, directly proportional to the degree of irradiation, when the 11 HGG cell line was used. For the same experimental conditions, when the 15 HGG cell line was used, the cytotoxic effect was not significantly augmented by the increase in radiation dose, for all three inhibitors used.

### 2.6. The Interaction between Combined Treatment with LY294002, PD 98059, and SP600125 and Ionizing Radiation in HGG Cell Lines

As can be seen in Table 3, in the 11 HGG cell line the combinations of SP600125 or PD 98059 with gamma-radiation resulted in both additive and synergistic effects while combined treatment with LY294002 and radiation resulted in an additive effect only.

When the 15 HGG cell line was used, all three inhibitors resulted in additive effect when combined with ionizing radiation (Table 3).

## 3. Discussion

Tyrosine kinase growth factor receptors and their signaling pathways have an important role in cancer cell growth and survival. Molecularly targeted agents inhibiting these membrane proteins have demonstrated cytotoxicity in several types of cancer cells, when tested in preclinical tumor models [13,14,26,27,28,29].

One such cell membrane receptor is the PDGFR, shown to play an important role in the growth and survival of many solid tumors, including HGG [30]. Actually, it was proven that the receptor is overexpressed in HGGs cell lines but also in tissue [5]. In the last years, various authors concluded that HGGs, especially glioblastomas, are emblematic for their heterogeneity which includes the expression of the PDGF family [31]. Additionally, the heterogeneity is present at two levels: intratumoral but also intertumoral. Studies performed by Jeremy N. Rich and collaborators [32] proved the existence of mixed clinical responses of anti-PDGFR-based strategies insisting on the necessity of models of cancers, in order to better develop new cancer therapies.

In our previous studies, we showed that both the level of expression and the response to its inhibition were different in various HGG-derived cell lines. We analyzed the effect of the PDGFR inactivation by AG1433 in different HGG immortalized cell cultures [13] but also in low passage HGG cell culture and the results were promising, encouraging us to continue the series of experiments. Therefore, we decided to extend our study on a broader cell panel, to better understand whether annihilation of the function of the receptor is beneficial in the treatment of HGG [16].

In this study, we found that the receptor was expressed in two HGG cell lines (11 and 15), the protein was found in elevated amount on the surface of 15 HGG cells while the 11 HGG cells presented lower levels of the receptor.

Our results were rather unexpected. We found that in 11 HGG cells that expressed the receptor less, the inhibitor reduced the viability of the cells in a dose and time-dependent manner. The AG1433 produced different effects on 15 HGG cells. We observed that the toxicity of the drug remained time-dependent (although less than in 11 HGG cells), but not in a dose dependent-manner.

In our previous studies evaluating the role of EGFR in the treatment of brain tumors, we also observed that 11 HGG cells expressed more EGFR at the cell surface compared to 15 cell culture, but receptor expression level was not correlated with the response to EGFR inhibition treatment [21]. In addition, the effect of insulin growth factor-1 receptor inhibition was also modest in 11 HGG cells [30]. One explanation of the GFRs inactivation failure in HGG treatment may be the signal transduction redundancy. It is well known that intracellular redundancy is an opposing phenomenon to cell sensitivity to GFRs inhibitors and this should be taken into account in finding better therapeutic solutions for these fatal tumors [33].

Even though the 11 HGG cell line responded better (50% at a concentration of 30 mM after 7 days of the treatment) than the 15 HGG cell line (33% at a concentration of 30 mM after 7 days of the treatment) to the treatment, we believe that in general, the response to the drug is promising and should be tested on the animal model for further clinical translation. We are, however, aware that the effect of PDGFR inhibition may be improved and given the demonstrated regulatory potential role of PDGFR in HGG cell response to radiotherapy, we also investigated the possibility to increase radiation response by blocking receptor activity. HGG actual standard of care consists of surgery followed by hyperfractionated radiotherapy associated with concurrent and adjuvant temozolomide. However, HGGs remain among the most aggressive human brain tumors determining the death of the patients in about 14.6 months [34]. Even if ionizing radiation serves as the most efficient therapy for HCG, this treatment approach continues to be only palliative, because of radioresistance. By using MTT assay, we previously found that 11 and 15 HGG cell lines are resistant to gamma radiation [21]. However, data from the literature showed that the most frequent method to analyze the response of cancer cells to radiation exposure is clonogenic assay, while MTT assay that has a single-point detection of cell proliferation is occasionally used. The clonogenic survival assay investigates cell capacity to proliferate endlessly, while maintaining its potential to give rise to a clone. In this case, the survival curve represents a relationship between the used radiation dose and the fraction of cells sparing their ability to form clones. Using the same irradiation configuration, in this study we analyzed the radiosensitivity of 11 and 15 cell cultures by clonogenic assay and in accordance with previous results; we found that both cell cultures were resistant to ionizing radiation.

Although the radiotherapy is an important therapeutic method for the patients diagnosed with HGGs there are several problems to be solved. One problem is the resistance to radiation therapy and the other is the dose and type of radiation. When we are talking about radio-resistance, it is known that there is the intrinsic radio-resistance of the cells, but also the brain stem tumor cells known to be radio-resistant but also capable to induce the radio-resistance [35,36]. The intrinsic radio-resistance of the cells is due to tumor heterogeneity and microenvironment [37]. Therefore, in the last years, the fight against tumor cells radio-resistance led to strategies such as the use of radio-sensitizing agents, administered either prior or during the radiotherapy. In this way it was hoped that the sensitivity of the tumor cells will be enhanced, and the near normal tissue will not be affected [38]. Moreover, concepts like synergy or supra-additivity were described [39].

The growth factors receptors are either overexpressed or overactivated in HGGs. For this matter, the association of radiation therapy with targeted therapy may be a response for the radio-sensitization of HGG cells [40].

Our previous results indicated that both the susceptibility towards PDGFR inactivation and the impact of the PDGFR inactivation on the radiation response were different in different HGG cell lines. Thus, in this study we decided to investigate the effects of AG1433, a PDGFR inhibitor, combined with γ-radiation in two other human HGG cell lines, which have proven to be very radioresistant.

In this study, for each cell line, a total of 30 different combinations, consisting of concomitant treatment with 10, 20, and 30 μM AG1433 and 2, 4, 6, 8, and 10 Gy radiation, were used. Unsuccessfully, the combined treatment did not result in a synergistic response in any cell lines studied. An additive effect was found in 50% of combinations for the 11 HGG cell line and 7% of combinations for 15 HGG. Unfortunately, this result is very unfavorable and does not encourage the use this combination of modalities, but as mentioned earlier, the response to therapy is different from one cell line to another, from one patient to another, and therefore it is important to analyze each separate case before deciding the therapeutic option. It is also possible that a better effect could be achieved by using an augmented dose radiation or an increased inhibitor concentration.

The exposure of tumor cells to radiation activates the intracellular signal transduction pathways. Growth factors and their receptors are also activated by radiation [41]. The effect of signal transduction inhibition fared better in comparison to PGDFR inactivation. The combination between three signal transduction inhibitors, LY294002 (PI3K inhibitor), PD 98059 (Erk1/2 inhibitor), and SP600125 (a selective inhibitor JNK1/2), and radiation managed to decrease viability of both cell lines when compared to monotherapy. The combination proved to be additive in 66.6% of the cases in the 11 HGG cell line and 33.3% synergic, while in the 15 HGG cell lines all of the combinations proved to be additive. What is surprising is the fact the while the addition of the drugs improved cytotoxicity, increasing the radiation dosage made no difference in one of the three combinations in the 11 HGG cell line and all of the combinations in the 15 HGG cell lines. This further underscores that radiation sensitivity is a multifaceted and complex process which requires further in-depth study.

In conclusion, the complex biology and intricate mechanisms which govern the HGG aggressive phenotype warrant a much broader study of how different major or minor drivers such as Tyrosine kinase inhibitors (TKIs) and the signaling pathways they activate can be involved in the development of new drugs. While several studies have shown the influence of PDGFR and the downstream intracellular signals are indeed implicated in gliomagenesis and its invasive pattern, there is no definitive consensus on the exact level of influence and how its inhibition can hinder tumor progression and treatment resistance. Our study shows that while the combination of receptor inhibition and radiotherapy did not yield encouraging results, signal transduction inhibition resulted in an increase in radiosensitivity.

## 4. Materials and Methods

### 4.1. Reagents

Anti-PDGFR and anti-actin rabbit polyclonal IgG antibodies were from Santa Cruz (Dallas, TX, USA); anti-rabbit and anti-mouse IgG horseradish peroxidase-linked antibodies and the ECI immunodetection reagents were purchased from Amersham Bioscience (Birmingham, UK); normal goat serum was purchased from Dako (Glostrup, Denmark), goat anti-rabbit fluorescein isothiocyanate FITC was purchased from Jackson ImmunoResearch Laboratories, Inc. (JIR) (West Grove, PA, USA); 4′,6-diamido-2-phenylindole (DAPI), Thyrphostine AG 1433 were purchased from Sigma (St. Louis, MO, USA). AG1433 was diluted in dimethyl sulfoxide (DMSO) to a stock concentration of 10 Mm and stored at 20 °C. The DMSO concentration was below 0.1% when the inhibitors were added in the cultured medium. MTT Cell Proliferation Kit was purchased from Roche Diagnostics GmbH (Mannheim Germany).

### 4.2. Cell Cultures and Treatment

The primary cell cultures 11 and 15 HGG that we used in the present study were established from tumors at Academic University Hospital in Uppsala, Uppsala, Sweden according to standard procedures. The cell lines have been previously characterized by Hagerstrand et al. [30]. The cells were cultured in Minimum Essential Medium (MEM) containing 10% fetal bovine serum (FBS), 2 mM glutamine, and antibiotic (100 IU/mL penicillin and 100 IU/mL streptomycin). The cells were grown in tissue culture flasks maintained in a 95% air/5% CO_2_ atmosphere at 37 °C in a humidified incubator. PDGFR, were inhibited by 10, 20, and 30 µM AG1433, for periods of times indicated in the figure legends. Additionallly, the appropriate control groups with diluents only were included.

### 4.3. Flow Cytometry

The human HGG cells were detached, washed with fluorescence-activated cell counting (FACS) buffer (PBS containing 3% FBS and 0.02% NaN3), blocked in 10% FBS, and incubated with anti-PDGFR at working dilution 1:10 for 40–45 min at 4 °C. The cells were then stained with FITC-conjugated second antibody for 30 min on ice. IgG2 isotype control Ab was added for each condition. Cells were analyzed using a FACS Calibur flow cytometer (BD Biosciences, Heidelberg Germany) and the CellQuest™ 3.3 software BD (San Jose, CA, USA). For each measurement, 100,000 events were acquired.

### 4.4. Irradiation

Cells were incubated in a 95% air/5% CO_2_ atmosphere 37 °C in a humidified incubator in culture medium until 70–80% confluence. Cells were then irradiated in a culture medium at room temperature with 2, 4, 6, 8, and 10 Gy, using a 137Cs radiation source. The ionizing radiation was given in a single-dose and the cell proliferation was analyzed, using the MTT assay.

### 4.5. MTT Assay

The cell proliferation was evaluated by MTT assay (Roche Diagnostic GmbH, Basel, Switzerland) that is based upon the cleavage of the yellow tetrazolium salt MTT to purple formazan crystals by metabolically active cells. A total of 1 × 10 cells/w/200 Ml medium were seeded in 96-well culture plates, incubated for 8 h and exposed to different concentrations of Tyrphostin. After 3 and 7 days of incubation, 10 mL of MTT labeling reagent were added to each well and plates were incubated at 37 °C for 4 h. Formazan products were solubilized and the optical density (OD) was measured at 595 nm. Results and relative cell viability were expressed as percentage of the untreated control cultures.

### 4.6. Clonogenic Assay

Cells were seeded in tissue culture flasks and incubated at 37 °C in a humidified incubator containing 5% CO_2_. Exponentially growing cells were irradiated with single doses of 0, 2, 4, 6, 8, or 10 Gy γ-irradiation, using a ^137^Cs source, Then, the cells were counted, diluted, plated on 60-mm culture dishes (100–7500 cells/60-mm dish) and incubated at 37 °C for 12 days. The plates were then fixed with 95% ethanol, stained with crystal violet, and washed twice with distilled water. Stained colonies containing more than 50 cells were counted. The values at every dose were divided by the control value for the survival fraction and plating efficiency and surviving fractions were determined for each cell line.

### 4.7. Surviving Fraction (SF) Determination

To calculate SF2 values, the plots of log SF vs. dose were matched to the linear-quadratic (LQ) model. In this model, the surviving fraction S of cells irradiated to a total dose *D* is given by the formula: ln*S* = −α*D* − β*D*^2^, where *S* is the SF and *D* is the radiation dose. The plating efficiency (PE) was determined as the percentage of the number of colonies observed to the number of cells seeded. The results were derived from two different experiments.

### 4.8. Statistical Analysis

Statistical comparison of mean values was performed using the Student’s *t*-test. Differences with a *p*-value of *p* < 0.05 were considered statistically significant. Interactions between drugs and radiation (I) were classified by the Multiplicative Method as follows: additive inhibition occurs when I1,2 = I1 + I2; synergism occurs when I1,2 > I1 + I2; and antagonism occurs when I1,2 < I1 + I2 [25].

## Figures and Tables

**Table 1:** Observed effects for the 11 HGG cell line.

Rad (Gy)	AG1433 (µM)	Days after the Treatment	Predicted Survival	Observed Survival	Effect
2	10	3	0.7	0.7	ADD
		7	0.4	0.5	SUB
	20	3	0.7	0.7	ADD
		7	0.3	0.4	SUB
	30	3	0.7	0.7	ADD
		7	0.3	0.4	SUB
4	10	3	0.7	0.7	ADD
		7	0.4	0.4	ADD
	20	3	0.7	0.7	ADD
		7	0.3	0.4	SUB
	30	3	0.7	0.7	ADD
		7	0.3	0.4	SUB
6	10	3	0.7	0.7	ADD
		7	0.3	0.4	SUB
	20	3	0.7	0.8	SUB
		7	0.3	0.4	SUB
	30	3	0.7	0.8	SUB
		7	0.2	0.4	SUB
8	10	3	0.7	0.7	ADD
		7	0.3	0.3	ADD
	20	3	0.7	0.7	ADD
		7	0.3	0.3	ADD
	30	3	0.6	0.8	SUB
		7	0.2	0.4	SUB
10	10	3	0.7	0.7	ADD
		7	0.3	0.3	ADD
	20	3	0.7	0.7	ADD
		7	0.2	0.3	SUB
	30	3	0.6	0.8	SUB
		7	0.2	0.4	SUB

**Table 2:** Observed effects for the 15 HGG cell line.

Rad (Gy)	AG1433 (µM)	Days after the Treatment	Predicted Survival	Observed Survival	Effect
2	10	3	0.6	0.7	SUB
		7	0.5	0.6	SUB
	20	3	0.6	0.7	SUB
		7	0.5	0.6	SUB
	30	3	0.6	0.7	SUB
		7	0.5	0.6	SUB
4	10	3	0.5	0.6	SUB
		7	0.4	0.4	ADD
	20	3	0,5	0.7	SUB
		7	0.4	0.5	SUB
	30	3	0.5	0.8	SUB
		7	0.4	0.5	SUB
6	10	3	0.5	0.7	SUB
		7	0.4	0.4	ADD
	20	3	0.6	0.7	SUB
		7	0.4	0.5	SUB
	30	3	0.6	0.8	SUB
		7	0.4	0.6	SUB
8	10	3	0.5	0.6	SUB
		7	0.3	0.4	SUB
	20	3	0.5	0.7	SUB
		7	0.3	0.7	SUB
	30	3	0.5	0.6	SUB
		7	0.3	0.5	SUB
10	10	3	0.5	0.6	SUB
		7	0.3	0.4	SUB
	20	3	0.5	0.8	SUB
		7	0.3	0.5	SUB
	30	3	0.5	0.7	SUB
		7	0.3	0.5	SUB

**Table 3:** Observed effects for cell lines 11 and 15 HGG.

Cell Line	Rad (Gy)	Type of Intracellular Signaling Inhibition	Predicted Survival	Observed Survival	Effect
11HGG	2	LY294002 10 µM	0.9	0.9	ADD
		PD 98059 10 µM	0.8	0.8	ADD
		SP600125 10 µM	0.6	0.5	SYN
	10	LY294002 10 µM	0.8	0.8	ADD
		PD 98059 10 µM	0.8	0.7	SYN
		SP600125 10 µM	0.6	0.6	ADD
15HGG	2	LY294002 10 µM	0.7	0.7	ADD
		PD 98059 10 µM	0.6	0.6	ADD
		SP600125 10 µM	0.3	0.3	ADD
	10	LY294002 10 µM	0.7	0.7	ADD
		PD 98059 10 µM	0.6	0.6	ADD
		SP600125 10 µM	0.3	0.3	ADD
